# Nutrient-dependent regulation of symbiotic nitrogen fixation in legumes

**DOI:** 10.1093/hr/uhae321

**Published:** 2024-11-26

**Authors:** Yanlin Ma, Chengbin Xiao, Jianquan Liu, Guangpeng Ren

**Affiliations:** State Key Laboratory of Herbage Improvement and Grassland Agro-Ecosystem, College of Ecology, Lanzhou University, No. 222 South Tianshui Road, Lanzhou 730000, China; MOE Key Laboratory of Cell Activities and Stress Adaptations, School of Life Sciences, Lanzhou University, No. 222 South Tianshui Road, Lanzhou 730000, China; State Key Laboratory of Herbage Improvement and Grassland Agro-Ecosystem, College of Ecology, Lanzhou University, No. 222 South Tianshui Road, Lanzhou 730000, China; State Key Laboratory of Herbage Improvement and Grassland Agro-Ecosystem, College of Ecology, Lanzhou University, No. 222 South Tianshui Road, Lanzhou 730000, China

## Abstract

Mineral nutrients are essential for plant growth and development, playing a critical role in the mutualistic symbiosis between legumes and rhizobia. Legumes have evolved intricate signaling pathways that respond to various mineral nutrients, selectively activating genes involved in nodulation and nutrient uptake during symbiotic nitrogen fixation (SNF). Key minerals, including nitrogen, calcium, and phosphorus, are vital throughout the SNF process, influencing signal recognition, nodule formation, the regulation of nodule numbers, and the prevention of nodule early senescence. Here, we review recent advancements in nutrient-dependent regulation of root nodule symbiosis, focusing on the systemic autoregulation of nodulation in nitrate-dependent symbiosis, the roles of nodule inception-like proteins, and the function of essential nutrients and their associated transporters in legume symbiosis. Additionally, we discuss several key research areas that require further exploration to deepen our understanding of nutrient-dependent mechanisms in SNF.

## Introduction

**Figure 1:**
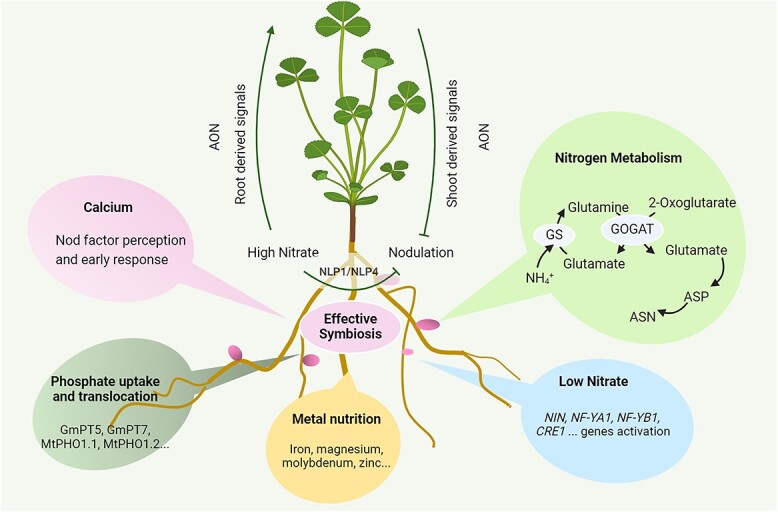
Overview of nutrient effects on SNF. Under low N conditions, rhizobium nodulation factors stimulate the expression of *NIN*, which in turn upregulates downstream genes like *NF-YA1*, *NF-YB1,* and *CRE1*, crucial for rhizobial infection and nodule formation. Calcium acts as a macronutrient in legumes, serving as a secondary messenger connecting nodulation factors perception at the plasma membrane with changes in calcium signaling. Legumes require high levels of inorganic phosphate for nodule growth, facilitated by phosphate uptake and translocation. The phosphate transporters GmPT5, GmPT7, MtPHO1.1, and MtPHO1.2 influence phosphorus uptake into nodules, subsequently affecting both nodule size and number. Macronutrients like sulfate and magnesium, along with micro-nutrients such as iron, molybdenum, and zinc, also play significant roles in nodule formation and N fixation. N metabolism is crucial for effective symbiosis, where ammonium produced through SNF is transported back to the plant and converted into glutamine and glutamate by GS and GOGAT. In indeterminate nodules, glutamine and glutamate are further converted into ASP and ASN. The AON system tightly regulates nodule number to balance N fixation with other developmental processes. This signaling pathway involves root-derived signals, shoot receptors, and shoot-derived inhibitors, enabling root-shoot-root communication to determine the optimal number of nodules. Abbreviations: ENOD40, early nodulation genes; NIN, nodule inception, NF-YA1, nuclear factor-Y subunit A-1, AON, autoregulation of nodulation. GS, glutamine synthetase. GOGAT, glutamate synthase. Images were created with BioRender (https://biorender.com).

Legumes interact with compatible rhizobia to form specialized root-derived organs, known as nodules, where atmosphere nitrogen (N_2_) is converted into ammonia (NH_3_) for use by the host plant. This process is called symbiotic nitrogen fixation (SNF). The beneficial symbiotic relationship between legumes and rhizobia revolves around the exchange of nutrients [1–3]. Plants provide carbon (C) in the form of carbohydrates produced through photosynthesis to the rhizobia, which in return fix N_2_ from the air for the plant [2, 4]. To effectively manage this symbiosis, host plants must tightly regulate SNF, including controlling the number of nodules, in order to balance their nitrogen (N) needs with C input [5, 6]. Legumes have developed sophisticated sensing and signaling systems to monitor environmental N levels and regulate SNF accordingly [7–9]. The autoregulation of nodulation (AON) pathway specifically controls nodule numbers in response to rhizobial infection. Additionally, nodule inception (NIN)-like proteins (NLPs) inhibit nodule formation in N-sufficient environments to conserve resources [10, 11] (Fig. 1).

In addition to C, plants supply mineral nutrients necessary for bacterial metabolism to their symbiotic partners [2, 12]. These mineral nutrients are delivered to rhizobia through roots, nodule vascular systems, and symbiosome membranes [2, 3, 13]. Transporters and enzymes play critical roles in this intricate nutrient exchange between nodules and host plants, as well as between host cells and symbionts. This is evidenced by the high expression levels of genes associated with metabolism and nutrient transport in mature nodules [14–16]. Effective SNF requires more than just a low-N environment. It also depends on calcium-mediated early symbiotic signal transduction between rhizobia and legumes, efficient phosphate uptake and translocation, the availability of various metal nutrients essential for plant and bacteroid growth and metabolism. Additionally, a precise N metabolism pathway is crucial for the success of the symbiotic relationship (Fig. 1).

In this review, we present an updated summary of recent advancements in understanding how essential mineral nutrients regulate SNF in legumes, building on existing knowledge. We explore the molecular mechanisms underlying nitrate regulation of nodulation, the roles of key nutrients in legume symbiosis, and the function of mineral nutrient transporters within root nodules. By elucidating the roles of critical regulators and their associated networks in nutrient-dependent SNF, this review aims to offer insights that could guide future research efforts in sustainable agricultural practices.

## Molecular mechanisms of AON in NO_3_^—^induced control of nodulation

**Figure 2:**
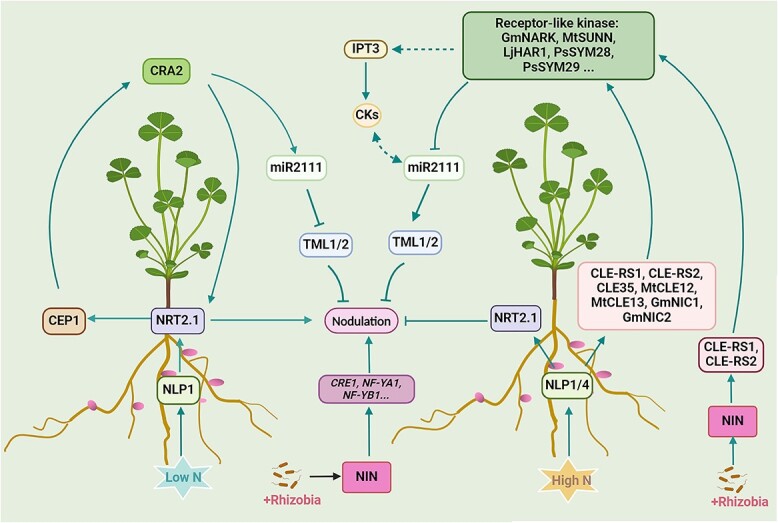
A general summarized model of rhizobia and NO_3_^−^ regulation in legumes: local and AON pathways for nodulation control. AON involves a long-distance signaling process that coordinates root and shoot responses. Under low N conditions, *MtCEP1* expression induces systemic *MtNRT2.1* expression through MtCRA2 in the shoot. MtNLP1 also activates low-level *MtNRT2.1* expression, further promoting nodulation and *MtCEP1* expression. NFs induce the activation of NIN, which upregulates genes essential for rhizobial infection and nodule formation, including *NF-YA1* and *CRE1*. Rhizobial infection activates *NIN* expression, leading to the synthesis of CLE-RS1 and RS2 peptides. Under high N conditions, MtNLP1 activates *MtNRT2.1* expression, increasing NO₃^−^ uptake and suppressing nodulation. MtNLP1 also activates *MtCLE35* and represses *MtCEP1*, systemically regulating nodulation. High NO_3_^−^ levels activate NRSYM1 (NLP4), inducing CLE-RS2 and RS3 peptide synthesis. CLE-like peptides (CLE-RS1, CLE-RS2, CLE35, MtCLE12, MtCLE13, GmNIC1, GmNIC2) are thought to be transported from root to shoot, where they are sensed by receptor-like kinases such as GmNARK, MtSUNN, LjHAR1, PsSYM28, and PsSYM29. The CLE-like peptides and receptor-like kinases form receptor-ligand complexes, reducing *miR2111* expression in the shoot. This decreases miR2111 levels in the root, increasing the transcript level of its target, *TML*. *TML* encodes an F-box protein that negatively regulates nodule formation on roots. CKs also act as shoot-derived inhibitors in the AON pathway. LjIPT3 is involved in CK biosynthesis in shoots and inhibits nodule formation, depending on the LjCLE-RS1/2-LjHAR1 signaling pathway. Images were created with BioRender (https://biorender.com)

The molecular mechanism of AON is a complex signaling system in legumes that regulates nodule formation to optimize N fixation and overall growth [6, 17, 18]. AON involves long-distance signals from developing nodules to the shoots and feedback from shoots to roots, which suppresses further nodulation [19, 20] (Figs 1 and 2). This system integrates both local and systemic signals to balance nodule numbers with environmental and nutritional conditions [1, 17, 21] (Fig. 2).

AON is mediated by CLAVATA3/ESR-related (CLE) peptides produced in response to rhizobia and high N levels [8, 22–27]. These peptides travel from roots to shoots via xylem, where they activate receptors that inhibit nodule formation through the shoot-derived inhibitor signal [28–31]. This process involves the CLE/SUNN-HAR1/miR2111/TOO MUCH LOVE (TML) network, which downregulates miR2111 and increases *TML* gene expression to limit nodules [22, 32, 33]. High N conditions further suppress nodulation via the NLP/CLE/SUNN pathway [8], while cytokinin (CK) produced in the shoot, regulated by the CLE-RS1/2-HAR1 module, also acts as a negative regulator of nodule formation [34] (Fig. 2). Additionally, the CRA2 pathway positively regulates nodules under low N, with CRA2 acting as a receptor for CEP1, integrating signals to maintain balance between lateral root and nodule formation [35–37] (Fig. 2). These findings underscore the complex interplay of systemic and local signals in controlling legume nodulation, ensuring that nodule numbers are finely tuned to environmental conditions and plant requirements.

## NLPs mediate nitrate inhibition of nodulation

RWP-RK domain-containing NLP transcription factors play a pivotal role in regulating nitrate-inhibited nodulation under high N conditions. In *Medicago truncatula*, NLP1 is essential for this process, directly binding to the *CLE35* promoter to activate its expression, thereby inhibiting nodulation [9]. Grafting experiments have further revealed the complex interaction between positive regulation by CRA2 and negative regulation by SUNN in the shoot, combined with NLP1’s inhibitory activity in the root. This interaction influences nitrate-induced rhizobial infection, nodule development, and nitrogenase activity, ultimately determining nodule numbers [9]. CRA2 positively regulates nodule formation by modulating the expression of *CEP1/2*, while further research indicates that NLP1 directly targets the *CEP1* promoter, suppressing *CEP1* expression in the presence of nitrate [38]. In addition to these findings, recent study has shown that in *Glycine max*, GmNLP1b and GmNLP4a bind to the promoters of nitrate-induced CLE peptide genes, *GmNIC1a* and *GmNIC1b*, activating their expression in response to nitrate and inhibiting nodulation [39]. These discoveries highlight the intricate interplay between systemic and local signals in nitrate-mediated nodulation inhibition. They also underscore the complex regulatory mechanisms through which NLPs mediate nitrate-induced inhibition of nodulation, demonstrating how legumes fine-tune their symbiotic interactions to adapt to varying N availability.

## The molecular mechanism of nitrate-induced nodule senescence

Nitrate-induced nodule senescence is another critical factor in the inhibition of SNF under high soil N conditions. Elevated nitrate levels suppress N fixation and disrupt nodule metabolism by reducing antioxidant content and nitrogenase activity, accelerating senescence [40, 41]. This process is marked by nodule color changes, increased reactive oxygen species, and damage to symbiosomes and bacteroids [42–45]. High nitrate also impairs carbon allocation to nodules and heightens oxygen diffusion resistance, further compromising nodule function [40, 46]. Although similar to natural senescence, nitrate-induced senescence progresses more rapidly, with its molecular pathways gradually being uncovered. The NAC-type transcription factor LjNAC094 has been identified as a key promoter of nitrate-induced root nodule senescence [47]. Acting downstream of LjNLP1 and LjNLP4, LjNAC094 regulates the expression of senescence-associated genes in response to nitrate [47]. Additionally, in soybean, nitrogen-associated NAPs (SNAPs) transcription factors SANP1/2/3/4 play critical roles in mediating nitrate-induced inhibition of nitrogenase activity and acceleration of nodule senescence [48]. These transcription factors reprogram the nodule transcriptome in response to N by directly regulating a subnetwork of senescence-associated genes and transcriptional regulators, including TFs in the NAC, WRKY, and ethylene responsive factor (ERF) families [48]. These findings elucidate the connection between nitrate signaling and nodule senescence progression.

## Essential nutrients and related transporters in the legume symbiosis

### Nitrogen

#### NO_3_^−^ transport

NO_3_^−^ and NH_4_^+^ are the primary inorganic N sources for plants, serving as essential nutrients and signaling molecules that regulate plant growth and metabolism [49, 50]. Nitrate transporters (NRT) from the NRT1 and NRT2 families enable root cells to uptake NO_3_^−^ through high- and low-affinity systems, depending on NO_3_^−^ availability [51, 52]. Key transporters like *Arabidopsis* NRT1.1 and *M. truncatula* NRT1.3 function as dual-affinity transporters, regulating NO_3_^−^ uptake based on soil NO_3_^−^ concentration [53, 54]. AtNRT1.1, the first plant NO_3_^−^ transporter identified, was initially linked to NO_3_^−^ uptake and transport [55]. Subsequent studies revealed that AtNRT1.1 can switch its NO_3_^−^ transport activity between high- and low-affinity states depending on its phosphorylation status [56, 57]. AtNRT1.1 serves as an NO_3_^−^ sensor, activating NO_3_^−^ response pathways independently of its NO_3_^−^ uptake function [56].

**Figure 3:**
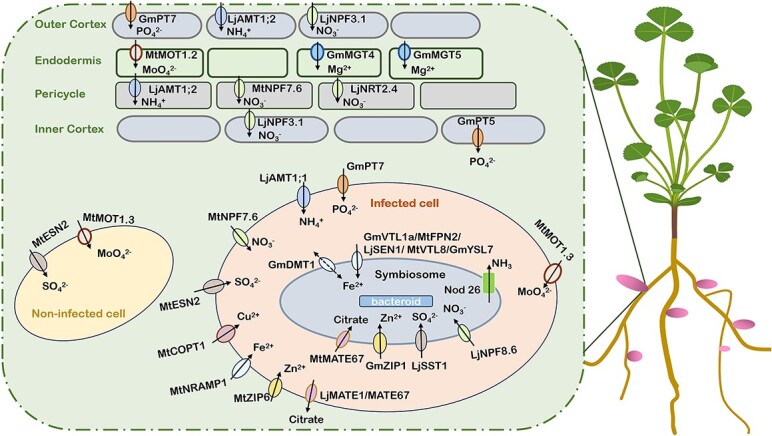
Major nutrient transporters for SNF in legume species. Upon reaching the infected cell, macro- and micronutrients must pass through the plasma membrane, the symbiosome membrane, and the bacteroid membrane in a specific sequence to supply essential resources for N fixation to the rhizobia. Upon arrival at the nodule, nutrients are extracted from the vasculature by specific transporters and transported into the endodermal cells. Subsequently, both macro- and micronutrients are released into the apoplast, where specialized transporters assist in their entry into nodule cells. Within the nodule cells, a network of transporters located on the symbiosome membrane directs the cytosolic nutrients towards the N-fixing bacteroids. Fully developed symbiosomes exhibit a variety of transporters on their membrane, as well as other crucial membrane proteins necessary for symbiosome membrane formation. Additional transporters can be found on the cell membrane and within the vasculature. It is important to acknowledge that although some transport systems are well-defined, not all of them are fully understood. Arrows indicate the direction of transport. Images were created with BioRender (https://biorender.com)

Several NRT1/peptide transporter family (NPF) proteins, such as MtNPF1.7 and LjNPF8.6, play significant roles in SNF in *M. truncatula* and *Lotus japonicus* [58–61]. MtNPF1.7, previously known as numerous infection and polyphenolics/lateral root-organ defective (NIP-LATD), regulates nodule meristem formation and invasion [60]. In *L. japonicus*, LjNPF8.6 also plays an active role in regulating nitrogenase activity and reactive oxygen species content in root nodules and exhibits dual-affinity NO_3_^−^ transport activity in oocytes [61] (Fig. 3). In *M. truncatula*, orthologs of AtNPF6.3/NRT1.1, namely MtNPF6.5 and MtNPF6.7 were identified. *MtNPF6.5* is repressed, while *MtNPF6.7* is induced by NO_3_^−^ in an MtNLP1-dependent manner, mediating NO_3_^−^ and chloride uptake [62]. MtNPF7.6, another critical transporter, functions in NO_3_^−^ uptake through nodule transfer cells to regulate nodule symbiosis [63]. Under NO_3_^−^ deficiency conditions, rhizobia induce *MtNPF7.6* gene expression in the vascular tissue of root nodules, facilitating NO_3_^−^ absorption and promoting nodule growth without competing with the host plant for NO_3_^−^ [63]. At low NO_3_^−^ levels, *MtNPF7.6* expression enables nodules to absorb NO_3_^−^ from the environment. However, excessive NO_3_^−^ inhibits leghemoglobin gene expression, disrupts NO homeostasis, and hinders nitrogenase activity, highlighting MtNPF7.6’s role in fine-tuning nodule development and SNF in response to fluctuating environmental NO_3_^−^ status [63]. LjNPF3.1, localized to the cortical regions of roots and nodules, facilitates NO_3_^−^ transport to the N-fixing zone within the nodule, playing a crucial role in nodule function under low N conditions [64] (Table 1, Fig. 3). The *ljnpf3.1* knockout mutants exhibit inhibited shoot growth and reduced anthocyanin accumulation due to nutrient deficiency [64]. Additionally, LjNRT2.4, a member of the *L. japonicus* NRT2 family, is vital for regulating NO_3_^−^ levels in nodule, optimizing nodule N-fixation activity [7]. Thus, understanding the regulatory roles of NO_3_^−^ transporters in nodule growth and SNF are crucial for optimizing N inputs in legumes throughout their development cycle.

**Table 1:** Summary of identified major nutrients transporters for SNF in different legume species.

**Transporter category**	**Name**	**Ion**	**Sublocalization**	**Reference**
Nitrate and ammonium transporter	LjNRT2.4	NO_3_^−^	PM	7
	Mt/LjNRT2.1	NO_3_^−^	PM	65, 66
	MtNRT1.3	NO_3_^−^	PM	53
	MtNPF7.6	NO_3_^−^	PM	63
	LjNPF3.1	NO_3_^−^	PM	64
	LjNPF8.6	NO_3_^−^	SM	7, 61
	LjAMT1;1	NH_4_^+^	PM	67
	LjAMT1;2	NH_4_^+^	PM	68, 69
Phosphorus	GmPT5	PO_4_^3−^	PM	70
	GmPT7	PO_4_^3−^	PM	71
	MtVPT2/3	PO_4_^3−^	unknow	72
	MtPHO1.1	PO_4_^3−^	PM/	73
	MtPHO1.2	PO_4_^3−^	PM/Golgi	73
Potassium	GmHAK5	K^+^	PM	74
	LjKUP	K^+^	PM	75
Magnesium	GmMGT4	Mg^2+^	PM	76
	GmMGT5	Mg^2+^	PM	76
Sulfate	LjSST1	SO_4_^2−^	SM	77
	MtESN2	SO_4_^2−^	PM	78
Iron	GmVTL1a	Fe^2+^	SM	79, 80
	MtFPN2	Fe^2+^	SM	81
	LjSEN1	Fe^2+^	SM	82
	MtVTL4/8	Fe^2+^	SM	83
	GmYSL7	Fe^2+^	SM	84–86
	MtYSL3	Fe^2+^	PM	87
	GmDMT1	Fe^2+^	SM	88
	MtNRAMP1	Fe^2+^	PM	89
Molybdenum	MtMOT1.2	MoO_4_^2−^	PM	90
	MtMOT1.3	MoO_4_^2−^	PM	91
Zinc	MtZIP1	Zn^2+^	SM	92
	MtZIP6	Zn^2+^	PM	93
	MtYSL3	Zn^2+^	PM	87
	MtMTP2	Zn^2+^	ER	94
Copper	MtCOPT1	Cu^2+^	PM	95
Citrate	LjMATE1	Citrate	PM	96
	LjMATE67	Citrate	PM/SM	97

Abbreviations: plasma membrane, PM; symbiosome membrane, SM; nuclear envelope, NE; endoplasmic reticulum, ER

Recent studies have elucidated the role of the NO_3_^−^ transport system in regulating nodulation and N fixation in high NO_3_^−^ conditions. Specifically, NRT2.1 has been identified as a key player, with its expression directly controlled by LjNLP1 in the presence of NO_3_^−^ in *L. japonicus* [65, 98]. Mutations in *LjNRT2.1* sustain nodulation in high NO_3_^−^ environments by weakening NO_3_^−^ induced inhibition. This mechanism involves intracellular NO_3_^−^ influx, nuclear translocation of LjNLP4, and regulation of symbiotic gene expression. Additionally, LjNIN counteracts the inhibitory effects of NO_3_^−^ on nodulation by competing with LjNLP1 for binding to the *LjNRT2.1* promoter, potentially reducing NO_3_^−^ uptake and facilitating nodulation [65, 98]. In *M. truncatula*, MtNRT2.1 has been revealed as essential for NO_3_^−^ uptake and optimal nodule formation under low NO_3_^−^ conditions. Studies indicate that *MtCEP1* is upregulated in low NO_3_^−^ environments, inducing *MtNRT2.1* expression via MtCRA2 in the shoot (Fig. 2). Furthermore, MtNLP1 actively stimulates *MtNRT2.1* expression, improving NO_3_^−^ uptake and promoting plant growth and nodulation [65, 66]. MtNLP1 enhances *MtNRT2.1* expression to increase NO_3_^−^ uptake while simultaneously suppressing nodulation and inhibiting *MtCEP1* expression. In summary, MtNRT2.1 is indispensable for effective nodule formation in low NO_3_^−^ environments and inhibits nodulation when NO_3_^−^ is sufficient [66] (Fig. 2). These discoveries shed light on how legumes adapt to N availability by modulating NO_3_^−^ uptake and transport, utilizing transcription factors to regulate N acquisition strategies.

#### N metabolism and transport

In legumes, atmospheric N_2_ is converted into NH_3_ by nitrogenase enzymes within the bacteroids of nodule cells. The resulting NH_4_^+^ is transported across cell membranes and converted into glutamine and glutamate via the glutamine synthetase (GS)-glutamate synthase (GOGAT) pathway [99]. This process begins when NH_4_^+^ binds to glutamic acid in an ATP-dependent reaction catalyzed by GS, forming glutamine [99] (Fig. 1). GOGAT then catalyzes the conversion of glutamine and 2-oxoglutarate to produce glutamic acid, completing the primary assimilation of ammonium [99] (Fig. 1). N export patterns vary with the host plant. Temperate legumes like *M. truncatula* and pea develop indeterminate nodules and mainly reduce N_2_ to amides such as glutamine and asparagine. Whereas, tropical legumes like *G. max* and *L. japonicus*, which form determinate nodules, use N_2_ to produce ureides like allantoin and allantoic acid, which are then transported to the stems and leaves through the nodule’s vascular system [100]. The ureides are essential for synthesizing critical biomolecules like amino acids, nucleotides, and chlorophylls. The suppression of *NADH-GOGAT* expression in *M. sativa* decreases nodule amino acid levels and negatively affects SNF [101]. Additionally, the enzyme glutamine phosphoribosyl pyrophosphate amidotransferase (PRAT) initiates ureide synthesis through purine oxidation, and inhibiting *PvPRAT3* reduces ureide production, impacting SNF [102]. *G. max* nodulin 26 (Nod26), a member of the aquaporin superfamily, is the major component of the symbiosome membrane (SM) enclosing N-fixing bacteroids in root nodules [103–105]. Nod26 promotes efficient N assimilation and prevents potential ammonia toxicity by binding to the conserved C-terminal domain of GS [106].

Ammonium is the main product of SNF. The transport and assimilation of NH_4_^+^ are critical processes in the plant-rhizobium interaction. *LjAMT1;1*, encoding a high-affinity NH_4_^+^ transporter, is expressed in both the infection zone and vascular tissue of *L. japonicus* nodules [67]. Inhibiting *LjAMT1;1* expression partially impairs N-fixing activity in nodules and increases nodule number compared to control plants, suggesting that LjAMT1;1 is involved in modulating NH_4_^+^ homeostasis in nodules [67]. In *G. max*, the transport of reduced allantoin and allantoic acid from nodules relies on a urea permease, UPS1 [107]. Repression of *GmUPS1–1* and *GmUPS1–2* expression in nodules leads to an accumulation of ureides and affects N translocation from nodules to shoots [107]. By expressing a common bean UPS1 transporter in the cortex and endodermis cells of soybean nodules, it was found that N transfer from nodules to the shoot and seed development were significantly increased [108]. Additionally, the number of transgenic nodules increased, and the nitrogenase activity per nodule was also enhanced, indicating that transporter function in N export from nodules is a key step for enhancing atmospheric N fixation and nodule function, as well as for improving shoot N nutrition and seed development in legumes [108].

### Phosphorus

Phosphorus (P), a vital component of nucleic acids, amino acids, phospholipids, and secondary metabolites, plays a crucial role in photosynthesis, energy conversion, and maintain enzyme activity, all of which are essential for plant growth and development [109, 110]. In legumes, the formation of nodules is an energy-intensive process that necessitates a significant amount of P [111–113] (Fig. 1). A deficiency in P can have a detrimental effect on nodule initiation, leading to a reduction in nodule number, size, and activity, ultimately hindering SNF [113, 114]. Adequate P supply has been shown to enhance *G. max* nodulation by increasing both nodule number and size [115]. Therefore, maintaining an appropriate level of P in nodules is crucial for overall plant growth and biological N fixation.

P acquisition in plants is mediated by P transporters. Inorganic phosphate (Pi) transport in rhizobia is crucial for efficient SNF. The regulation of P transporters is essential for plant adaptation to low-P stress [116, 117]. The P transporter family protein PHT1/PT is primarily responsible for the uptake of Pi from the soil and its mobilization within plants, maintaining Pi homeostasis [117, 118]. The *G. max* gene *GmPT5* encodes a high-affinity phosphate transporter that transports Pi from roots to nodules, particularly under limited P conditions [70] (Figs 1 and 3). Another nodule-localized phosphate transporter, GmPT7, absorbs Pi from the external environment and transfers it from the nodule cortex to the fixation zone, participating in SNF in legumes [71] (Fig. 3). Similarly, over-expression of the rice (*O. sativa*) phosphate transporter gene *OsPT2* in *G. max* enhances both N fixation and NH_4_^+^ assimilation under P-deficiency conditions [119]. Vacuolar phosphate transporters (VPTs) modulate P adaptation and play essential roles in mutualistic rhizobium–legume symbiosis by regulating long-distance Pi transport. Mutations in *MtVPT2* and *MtVPT3* result in cytosolic Pi deficiency in nodules, reducing nodule number and nitrogenase activity under different phosphate conditions [72]. Therefore, MtVPT2 and MtVPT3 are crucial for maintaining a stable cytosolic Pi level in the fixation zone of the nodule under low-phosphate stress, as well as for regulating nitrogenase activity and phosphate homeostasis in root nodules [72].

Phosphate starvation response 1 (PHR1), a constitutively expressed MYB-domain transcription factor, binds to a *cis*-element in its target genes, regulating their expression and promoting Pi uptake by directly inducing the expression of *PHOSPHATE-TRANSPORTER1* (*PHT1*) [120, 121]. The PHR1-PHT1 module is a key regulatory component in the plant P signaling pathway [118]. Multiple GmPHR-GmPHT1 modules operate in both infected and noninfected nodule tissues, with each GmPHR targeting multiple GmPHT1s and vice versa. These overlapping modules regulate Pi homeostasis, nodule initiation, and development. Over-expression of *GmPHR1* boosts *GmPHT1;11* expression, increasing nodule Pi content and size, underscoring the importance of the PHR-PHT1 module in nodules [122].

Proteins related to Pi homeostasis, such as GmSPX8, GmSPX5, MtPHO1.1, and MtPHO1.2, are essential for *G. max* nodulation. GmSPX8, a nucleus-localized SPX protein, is vital for nodule development and N fixation under low P conditions [123]. Overexpression of *GmSPX8* increases nodule number, weight, and nitrogenase activity, enhancing N and P content in P-deficient environments, whereas suppression impairs these processes [123]. GmSPX8 interacts with GmPTF1, further promoting nodule development and N fixation [123]. Both *GmSPX5* and *GmSPX8* exhibit a predominant expression in nodules and show an increase in expression levels when phosphorus (P) deficiency occurs. The overexpression of either of these proteins leads to an increase in both the number and weight of nodules, which in turn results in enhanced N and P content within the nodules [123, 124]. GmNF-YC4, which is a transcription factor belonging to the nuclear factor Y family, interacts with GmSPX5, enhancing its binding to downstream gene promoters and supporting nodule development and function [124]. In *M. truncatula*, the PHO1 family members MtPHO1.1 and MtPHO1.2, found in the plasma membrane and Golgi, respectively (Table 1, Fig. 3), play a vital role in the transport of Pi from the infected nodule cells to the bacteroids [73].

### Potassium

Potassium (K) plays a vital role in plant responses to abiotic stress, particularly in regulating osmotic pressure, turgor, and ion homeostasis [125–127]. K transport is crucial during root nodule formation and development, with nodules showing heightened sensitivity to ionic stress, likely due to ion homeostasis disruptions in infected cells. In *M. truncatula*, studies reveal significant declines in K levels within symbiosomes and vacuoles of infected cells over the cell life cycle, potentially disturbing K homeostasis [128]. Low-temperature scanning electron microscopy and X-ray microanalysis highlight substantial reductions in K during nodule maturation and senescence, correlating with the mislocalization or partial loss of key Shaker K channels, MtAKT1 and MtSKOR/GORK, impairing K balance and nodule function [128]. K availability is also critical for SNF, as it enhances ATP production and electron flow, thereby supporting N fixation [129]. Additionally, K mediates the transfer of carbohydrates from the host plant to the nodules, modulating C input and N output, which further affects the N fixation process [2, 129, 130]. In *L. japonicus*, the K transporter *LjKUP*, highly expressed during late nodule development, is localized to the plasma membrane [75]. Similarly, in soybean, the K transporter *GmHAK5*, highly expressed in vascular tissues of roots and nodules, facilitates K translocation from roots to nodules [74]. Therefore, K is essential for nodule development, ion homeostasis, and efficient SNF in legumes.

### Calcium

Calcium (Ca) is integral to plant nutrition and signal transduction, functioning as a key component of cell walls and membranes, as well as an intracellular second messenger [131]. In legume–rhizobia symbiosis, Ca is crucial throughout preinfection, infection, and nodule development [132–134]. Ca deficiency, particularly in acidic soils, disrupts nodule formation and growth by impairing rhizobial multiplication and root infection [135, 136]. The interaction between Ca and boron under stress conditions enhances SNF efficiency [137]. Additionally, Ca acts as a secondary messenger in Nod factor signaling, modulating gene expression during early symbiotic events [133, 138, 139]. Ca^2+^-ATPases and Ca-dependent protein kinases are essential for active Ca transport and regulation within the symbiosome, underscoring its critical role in nodulation and SNF [140, 141].

### Sulfur

Sulfur (S) is the fourth essential nutrient element for plants after N, P, and K. It is a component of the amino acids cysteine and methionine, glutathione, plant chelating agents, coenzyme A, and other S compounds, and it participates in important physiological and biochemical reactions in plants [142, 143]. In legumes, S supply is positively correlated with SNF, and S deficiency can cause three additional main effects: fewer nodules, inhibition of SNF, and inhibition of nodule metabolism [144, 145]. Sulfate transporters facilitate the absorption and transport of sulfate [143]. In *L. japonicus*, the sulfate transporter SST1 is located on the SM and mediates sulfate transport from the plant cell cytoplasm to intracellular rhizobia [2, 77]. Additionally, *Early Senescent Nodule 2* (*MtESN2*) in *M. truncatula* encodes a protein of the sulfate transporters family, expressed in both roots and nodules. This protein localizes to the plasma membranes of infected and uninfected cells within the transition and N-fixing zones of nodules [78] (Fig. 3). The *Mtesn2* mutant exhibits significantly reduced nodule size and nitrogenase activity, with nodules showing early senescence under symbiotic conditions [78].

### Iron

Iron (Fe) is critical for SNF due to its role in Fe-dependent proteins, including leghemoglobin, ferroproteins, nitrogenase, cytochrome, and hydrogenases [146–150]. Fe is required at all stages of nodulation and SNF in legumes, though its impact varies by species [149]. In *Lupinus angustifolius*, Fe deficiency impairs both nodule initiation and development, whereas in crops like soybean, peanut, and common bean, it primarily affects later stages of nodule maturation [148, 151]. The requirement for Fe in legume–rhizobia symbiosis is higher than in nonleguminous plants, and Fe deficiency reduces nodule number, size, and N fixation efficiency [149, 152, 153]. In *Phaseolus vulgaris*, nodule Fe concentration correlates positively with N fixation rates [152]. To compensate for Fe deficiency, soybean initiates adaptive responses such as increased H^+^ secretion and Fe(III) reductase activity to enhance Fe uptake from the soil [154].

Typically imported into the nodule as ferric citrate via the xylem, Fe must then traverse several cell layers to reach the infected cells [147, 155]. This transport occurs through both symplastic and apoplastic routes [155]. Specifically, two multidrug and toxic compound extrusion (MATE) transporters LjMATE1 and MtMATE67 play important roles in delivering Fe to the nodule infection zone, thus maintaining Fe homeostasis in nodules [96, 97]. Additionally, MtNRAMP1, an Fe transporter in the cytoplasmic membranes of infected root nodule cells, facilitates apoplastic Fe uptake, promoting SNF [89] (Fig. 3). Moreover, nodule-specific vacuolar iron transporter-like 4 (MtVTL4) and MtVTL8, particularly MtVTL8, the closest homolog to LjSEN1, serve as main Fe transport proteins to bacteroids in *M. truncatula* [83] (Fig. 3). In *G. max*, the GmVTL1a transporter functions in the N fixation region of root nodules, transporting Fe across the symbiotic membrane to bacteroids [79] (Fig. 3). Furthermore, ferrous Fe uptake by infected cells is facilitated by the nodule-specific protein Ferroportin2 (MtFPN2) in *M. truncatula*, located in symbiosomes in the interzone and early-fixation zone, delivering Fe to N-fixing bacteroids [81]. Similarly, *GmDMT1* (*G. max divalent metal transporter 1*), a member of the NRAMP/Dmt1 family, is highly expressed during the initial stages of N fixation in developing *G. max* nodules. It functions as a nodule-enhanced divalent metal transporter, facilitating the transport of ferrous Fe across the peribacteroid membrane [88]. The yellow stripe-like (YSL) family of metal transporters is essential for transporting Fe from roots to nodules. For instance, MtYSL3, located in the plasma membranes of vascular cells in roots and nodules, as well as cortical nodule cells, facilitates the delivery of Fe and Zn to N-fixing nodule cells, playing a critical role in SNF [87]. Recent research has highlighted the roles of YSL7 proteins in symbiotic nodulation in legumes [3, 84–86]. Specifically, MtYSL7, a clade III YSL protein, localizes to the plasma membranes of cells within the root pericycle, nodule cortex, and vasculature [85] (Fig. 3). It functions in oligopeptides transport, thereby maintaining iron (Fe) and copper (Cu) homeostasis in nodules, which is crucial for SNF [85]. Similarly, GmYSL7 in *G. max*, specifically situated in the SM, regulates nodule development and nitrogenase activity by transporting oligopeptides to symbiosomes [86]. Additionally, *GmYSL7*, highly expressed in multiple nodule cells, responds to changes in Fe levels, facilitating Fe accumulation in *G. max* nodules, thereby influencing nodule development and N fixation activity [84].

### Magnesium

Magnesium (Mg), the most abundant divalent cation in cells, is crucial as an activator of numerous enzymes and is essential for various physiological processes in plants, including photosynthesis, carbon metabolism, and protein synthesis [156, 157]. During SNF, the rhizobium nitrogenase relies on Mg-ATP hydrolysis to transfer electrons from the Fe protein to the Mo–Fe protein [158]. While Mg does not directly affect nodule formation, it influences nodule development by optimizing carbohydrate distribution and facilitating the transfer of carbohydrates from shoots to nodules. Mg specifically regulates carbon and N transport and exchange within nodules [3, 159]. Specifically, Mg accumulation in the inner cortex of *G. max* nodules activates the activity of the β-1,3-glucanase GmBG2 in inner cortical cells, leading to callose decomposition, increased plasmodesmata permeability, and enhanced carbon-N exchange [76]. Leguminous plants transport Mg to nodules either by unloading it from vascular tissues or by directly absorbing it through the nodule cortex. GmMGT4 and GmMGT5, members of the *G. max* Mg Transporter (MGT) family located on the plasma membrane of the inner cortex within the nodule, together facilitate Mg absorption and accumulation in cortical cells [76] (Fig. 3). Disruption of *GmMGT4* and *GmMGT5* inhibits nodule growth, increases callose accumulation in cortical cells, decreases sugar content, and reduces N export rates, highlighting their critical roles in maintaining Mg homeostasis and supporting effective symbiosis [76].

### Molybdenum

Effective SNF require a steady supply of molybdenum (Mo), a component of the Fe–Mo cofactor of nitrogenase [160, 161]. Mo plays a crucial role in molybdoenzymes such as nitrogenase, xanthine dehydrogenase, and nitrate reductase, facilitating electron transfer during SNF [162]. In *Bradyrhizobium japonicum*, the high-affinity ModABC transport system mediates molybdate uptake, essential for efficient N fixation, indicating molybdate transport across the symbiosome membrane [163]. Additionally, molybdate is required for the Mo cofactor (Moco), which supports essential metabolic processes, including nitrate assimilation, purine metabolism, and sulfite detoxification [160, 162, 164]. In *M. truncatula*, two Mo transporters, MtMOT1.2 and MtMOT1.3, are crucial for Mo uptake into nodules [90, 91]. MtMOT1.2 is located in the plasma membrane of endodermal cells surrounding vascular vessels in nodules, facilitating Mo uptake and distribution within nodule cells [90] (Fig. 3). MtMOT1.3, located in the plasma membranes of infected cells, transports MoO_4_^2−^ into nodule cells, essential for synthesizing functional nitrogenase [91] (Fig. 3).

**Figure 4:**
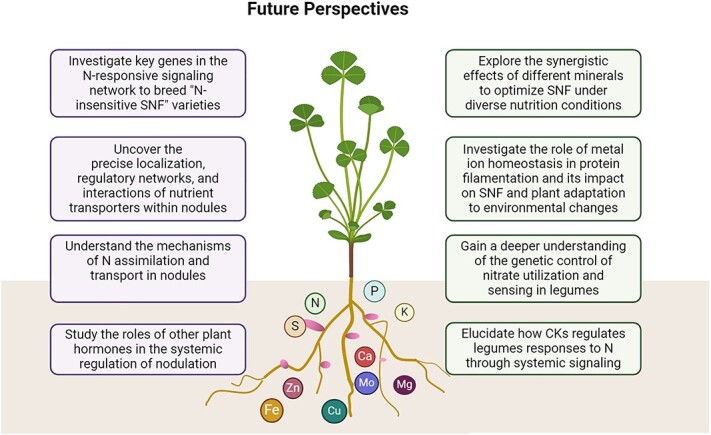
Outlooks in enhancing N Fixation and nutrient uptake in legumes

### Zinc

Zinc (Zn) is essential for numerous proteins and enzymes, playing key roles in chlorophyll biosynthesis and auxin production [3]. It stabilizes zinc-finger transcription factors, ensuring accurate DNA interactions for gene regulation [165, 166]. In legume root nodules, Zn is integral to homocitrate synthase, a metalloenzyme responsible for homocitrate production, a precursor in the formation of the iron–molybdenum cofactor of nitrogenase, which is crucial for SNF [2, 167]. Since most rhizobia lack the ability to synthesize homocitrate, they rely on their host plants for its production via the *FEN1* gene [167]. Additionally, Zn supports Cu–Zn superoxide dismutase, an enzyme that reduces oxidative stress in nodules, preserving nodule health and nitrogenase activity [3, 168]. GmZIP1, a Zn transporter specific to *G.max* symbiosis, is located in the peribacteroid membranes of root nodule cells, aiding in Zn uptake within symbiosomes [92] (Fig. 3). Conversely, MtZIP6, located in the plasma membranes of N-fixing nodule cells, facilitates Zn uptake from the apoplast [93] (Fig. 3). Additionally, MtMTP2, an intracellular Zn efflux transporter, regulates nodule development and bacteroid differentiation [94]. Collectively, MtZIP6 and MtMTP2 are critical to the maintenance of Zn homeostasis in nodules, constituting integral components of the Zn transport system essential for effective SNF. Recently, a pivotal study has elucidated the role of Zn as an intracellular second messenger, mediating the conversion of soil N nutrients into Zn concentration signals within root nodules [169]. This process is integral for linking environmental changes to N homeostasis through the regulation of the transcriptional regulator FIXATION UNDER NITRATE (FUN). Zn modulates the transition of FUN from an inactive filamentous megastructure to an active transcriptional regulator, thereby optimizing nodule function in response to varying N conditions [169]. Under low N conditions, elevated Zn levels within nodules promote the formation of inactive FUN filaments, facilitating efficient N fixation. Conversely, in high N environments, decreased Zn concentrations trigger the dissociation of these filaments and activation of FUN, orchestrating multiple pathways to suppress N fixation and induce nodule senescence [169]. This mechanism highlights the potential of FUN in enhancing legume tolerance to soil NO_3_^−^, improving the efficiency of fixed N delivery, minimizing fertilizer dependence, and advancing sustainable agricultural practices.

### Copper

Copper (Cu) is critical for electron transfer in photosynthesis, respiration, and carbohydrate metabolism, as well as regulating free radicals through Cu–Zn superoxide dismutase [3, 170]. In legume nodules, Cu is essential for cytochrome *cbb*_3_ oxidase activity, facilitating the microaerobic respiration of rhizobial bacteroids, a process vital for SNF [2, 171, 172]. Additionally, Cu supports bacterial tyrosinase, which is important for rhizobial survival [3, 173]. Copper transport from host cells to bacteroids is mediated by nodule-specific transporters like MtCOPT1 [95], while copper chaperones such as NCC1 enhance intracellular Cu delivery [174], ensuring the functionality of N-fixing enzymes like cytochrome oxidase and nitrogenase.

## Influence of soil nutrient availability and pH on N fixation in legumes

N fixation in legumes is significantly influenced by soil nutrient availability and pH. In P-deficient soils, P fertilization enhances N fixation by promoting root growth and extensive nodule development [175]. However, low soil pH negatively affects rhizobial survival and activity, reducing N fixation efficiency [176, 177]. Acidic conditions also increase the solubility of toxic elements like aluminum and manganese, which further inhibit root growth and nodule formation [178]. Legumes perform best in soils with a pH between 6.0 and 7.5; when pH drops below 5.5, the availability of essential nutrients such as Ca, Mg, and P decreases, adversely affecting nodulation and N fixation [176, 179]. Liming is often used in acidic soils to raise pH and improve nutrient availability, creating more favorable conditions for N fixation [180]. Mo deficiency, exacerbated in acidic environments, can further impair N fixation due to its role in the nitrogenase enzyme complex [160, 181]. The distribution and abundance of soil nutrients directly regulate N fixation by affecting nodule formation, rhizobial activity, and plant health. Nutrient deficiencies and unfavorable soil conditions, such as low pH or poor aeration, substantially hinder N fixation, while well-balanced nutrition and optimal environmental conditions support robust legume–rhizobia symbiosis, maximizing N fixation [182, 183]. Understanding these dynamics is crucial for optimizing N fixation in legume-based agriculture.

## Conclusion and future directions

Mineral nutrients are essential for the symbiosis between legumes and rhizobia. This review examines recent research advancements in the complex signaling pathways that respond to these nutrients, activating genes involved in nodulation and nutrient uptake during SNF. Findings indicate that the efficiency of SNF in legume root nodules, facilitated by rhizobia, is influenced by the host plant’s genetic background and the availability of essential mineral nutrients (N, P, K, Ca, Mg, S, Fe, Zn, Cu, Mo). Each nutrient plays a unique role in N fixation mechanisms. While significant progress has been made in understanding their individual contributions, comprehensive knowledge of their coordinated regulation is still lacking. Future research should focus on exploring the synergistic effects of these minerals and developing integrated strategies to optimize SNF under various environmental conditions (Fig. 4). We propose two key research directions related to mineral nutrients that require further enhancement:

(1) Investigating nitrate and hormonal interactions

N availability, particularly in the forms of nitrate (NO_3_^−^) and ammonium (NH_4_^+^), critically modulates SNF efficiency. Low NO_3_^−^ levels promote SNF, whereas high levels inhibit it [6, 184]. Specific transporters from the NRT1 and NRT2 families regulate nitrate uptake and distribution, directly influencing SNF [16, 65, 185]. Understanding the genetic control of NO_3_^−^ utilization and sensing in legumes is essential for improving biological N fixation and NUE. Additionally, further exploration is needed regarding the role of plant hormones, such as CKs, in regulating nodulation and responses to N and C levels in conjunction with mineral nutrients (Fig. 4).

(2) Enhancing molecular understanding of legume–rhizobia symbiosis

Rhizobia induce nodule formation and differentiate into bacteroids, which convert atmospheric N_2_ into NH_3_. However, the metabolic mechanisms controlling C and N allocation in bacteroids involving in mineral nutrients are not fully understood. Insights into the molecular mechanisms of fixed N transport and assimilation that are related to mineral nutrients could enhance the effectiveness of legume–rhizobia symbiosis. Recent advancements in single-cell RNA sequencing and proteomics offer new opportunities to investigate nutrient transporter functions. Furthermore, developing ‘N-insensitive SNF’ leguminous crop varieties could sustain high N fixation efficiency under heavy fertilization, mitigating the inhibitory effects of excess N on nodulation and SNF (Fig. 4).

In summary, while significant progress has been made in understanding the molecular mechanisms underlying SNF and nutrient transport in legumes, the regulatory networks governing these processes require further investigation. Focusing on nutrient transporters and signaling pathways will be crucial for advancing our knowledge of legume–rhizobia symbiosis and improving SNF efficiency in sustainable agriculture.

## Data Availability

All data supporting the findings of this review are available within the article.
